# Evaluation of the Technicon Star system for the estimation of serum T_4_ and T_3_-uptake

**DOI:** 10.1155/S1463924681000576

**Published:** 1981

**Authors:** S. G. Welshman, M. D. McMaster, T. Linton

**Affiliations:** Clinical Chemistry Department The Laboratories Belfast City Hospital Belfast Ireland


calculations were carried out with log $K_1$ and log $K_2$ values taken from the literature [10], then repeated with new values of 0.1 units larger and smaller than the original ones. Thus, the $V_a$ values were calculated from the data points in four different cases and averaged individually. Using the four average values four titration curves were calculated along with the sums of the squares of the deviations from the experimentally obtained ones. In a second step log $K_1$, or log $K_2$ was changed in such a manner that the sum of the squares became lower. If the sum of the squares was found to be greater again, the calculation was returned to the former step. The cycles were repeated with new log $K_1$ and log $K_2$ values until the minimum value for the sum of the squares was achieved. The log $K_1$ and log $K_2$ values found were 3.47 and 4.55 respectively. The calculated quantities of formic and acetic acids are given in Table 1. It can be seen that the accuracy of the determination – considering the fundamental difficulties – is acceptable.

Using the iteration method, there is no need for the extra titration procedures of the single acids and for the determination of the constants. This is especially advantageous when the ionic strength of the sample solution is not known. As a matter of fact, when the titrations of the single acids are carried out at different ionic strengths an uncontrollable error can be introduced into the calculations.

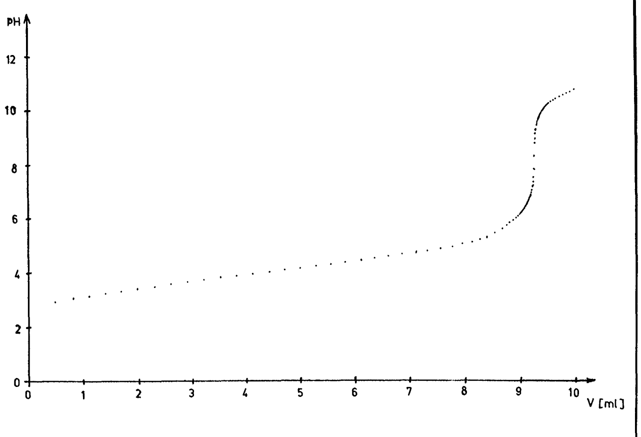

*Figure 4. Titration curve of a solution containing formic and acetic acid. Titrant: carbonate free potassium hydroxide: 0.1083 mol/dm³*
$V_m = 9.29$ ml; $V_a = 4.45$ ml

REFERENCES

[1] Johansson, A. and Johansson, S. (1978), *Analyst,* **103**, 305-316

[2] Johansson, A. and Johansson, S. (1979), *Analyst,* **104**, 601-612

[3] Leggett, D. J. (1978), *Anal Chem,* **50**, 718

[4] Firstenberg, S. and Malmvig, H. (1978), *American Laboratory,* 2, 119-122

[5] Betteridge, D., Dagless, E. L. and David, P. (1976), *Analyst,* **101**, 409-420

[6] Nowogrocki, G., Cannone, J. and Wozniak, M. (1979), *Anal Chim Acta,* **112**, 185-192

[7] Gampp, H., Maeder, M., Zuberbukler, A. D. and Kaden, T. A. (1980), *Talanta,* **27**, 313-318

[8] Ivaska, A. (1974), *Talanta,* **21**, 1167-1173

[9] Ivaska, A. and Nagypal, I. (1980), *Magy Kem Foly,* **86**, 2, 84-88

[10] Inczédy, J. (1976), Analytical Application of Complex Equilibria. Ellis Horwood, Chichester

### Evaluation of the Technicon Star system for the estimation of serum $T_4$ and $T_3$-uptake

**S. G. Welshman, M. D. McMaster and T. Linton**
*Clinical Chemistry Department, The Laboratories, Belfast City Hospital, Belfast, Northern Ireland.*

A Technicon Star system supplied by Technicon Instruments Ltd, (Basingstoke, Hampshire, England) was installed to provide a fully automated process for the estimation of serum thyroxine ($T_4$) and $T_3$ -uptake by radioimmunoassay (RIA). The $T_3$-uptake test is a technique used for assessing unsaturated thyroxine-binding globulin capacity in serum by measuring the uptake of radioactive tri-iodothyronine ($T_3$). A daily workload of approximately 150 serum $T_4$ assays and a similar number of $T_3$-uptake tests had previously been estimated by manual radioimmunoassay techniques. An evaluation of the Star system for the estimation of serum $T_4$ and $T_3$-uptake is described.

### Technicon Star system

A continuous flow analyser with computerised control and print-out was used for the automation of radioimmunoassays. The Star RIA system involves competition for antibody sites in the solid-phase reagent between the radio-labelled antigen and the antigen in the sample. The amount of radio-labelled antigen bound to the solid-phase reagent is inversely proportional to the amount of antigen in the sample. As the solid-phase reagent contains ferric oxide particles, the solid-phase bound antigen is retained by activated magnets and the unbound antigen removed to waste. When the magnetic field is removed, the bound radio-labelled antigen is released for measurement in a gamma counter. As the analyser is on-line to a laboratory computer, the results of both $T_4$ and $T_3$-uptake tests on each serum sample are fed directly to the computer which then calculates the free thyroxine index (FTI) and prints the final report. The reagents for these analyses were originally purchased from Technia, however the company has ceased to operate and all kits are now supplied by Technicon. All results referred to in this paper were obtained using the latter kits.

### The system in operation

The analyser processes samples at the rate of 60 specimens per hour. Starting-up time was approximately 30 minutes and change-over time between different assays was 15 minutes. It was found that one operator could process 150 $T_4$ and $T_3$-uptake tests in 6 hours. During processing only intermittent attention was required for the purpose of changing sample plates.

The Star system gave considerable trouble in the first few months following installation until it was discovered that the analyser was connected to an overloaded electrical circuit. The system was then put on a lightly-used circuit and has now been running for almost 9 months virtually trouble free. A Technicon engineer was called to replace a faulty flow monitor and a printer switch. The analyser required partial retubing monthly and a complete retubing every 3 months. This maintenance involves about one man-hour.

### Evaluation of the $T_4$ assay

### Accuracy and recovery

Sera containing low, medium and high concentrations of $T_4$ were prepared by adding known amounts of thyroxine to a batch of thyroxine-free serum. In each case the serum was analysed 20 times. The total amount of $T_4$ recovered and the percentage recovery are shown in Table 1.

### Precision

To determine the within-batch precision for $T_4$, sera containing low, medium and high concentrations of $T_4$ were each analysed 20 times. The between-batch precision was obtained from the estimation on each serum in 20 separate batches. Results are given in Table 2.

### Carryover and drift

Triplicate samples of serum containing 250 nmol/l of $T_4$ alternating with triplicate samples of serum with a value of 22 nmol/l were run continuously for 5 hours. No significant carry-over or drift was observed during this period.

**Table 1. Accuracy and recovery of $T_4$**

| No. | $T_4$ added (nmol/l) | Mean $T_4$ measured (nmol/l) | % Recovery ± SD |
|---|---|---|---|
| 20 | 51.0 | 51.5 | 101.0 ± 5.1 |
| 20 | 129.0 | 135.3 | 104.9 ± 4.2 |
| 20 | 257.0 | 275.6 | 107.2 ± 5.6 |

**Table 2. Precision of $T_4$ assay**

| Within-batch | | | Between-batch | | |
|---|---|---|---|---|---|
| Mean $T_4$ (nmol/l) | SD (nmol/l) | CV (%) | Mean $T_4$ (nmol/l) | SD (nmol/l) | CV (%) |
| 21.9 | 1.5 | 6.8 | 23.6 | 2.6 | 11.1 |
| 99.7 | 3.1 | 3.1 | 98.3 | 6.0 | 6.1 |
| 248.9 | 9.4 | 3.8 | 248.6 | 13.1 | 5.3 |

**Table 3. Precision of $T_3$-uptake assay**

| Within-batch | | | Between-batch | | |
|---|---|---|---|---|---|
| Mean $T_3$-U (%) | SD (%) | CV (%) | Mean $T_3$-U (%) | SD (%) | CV (%) |
| 31.2 | 0.9 | 2.9 | 31.4 | 1.1 | 3.4 |
| 39.1 | 0.6 | 1.5 | 39.4 | 1.0 | 2.5 |
| 56.3 | 0.6 | 1.1 | 56.9 | 1.3 | 2.3 |

### Normal $T_4$ values

A normal range was determined by analysing sera from 309 healthy adult blood donors. The mean $T_4$ value was found to be 100.8 nmol/l with a standard deviation of 20.3 nmol/l. The distribution was Gaussian.

### Correlation with manual method

A correlation between the Star $T_4$ system and the manual Amersham $T_4$ (PEG) assay supplied by Amersham International, (Amersham, England) was determined on 90 serum samples with values from 4 to 268 nmol/l. A coefficient of correlation of 0.95 and a linear regression equation $Y = 0.95X + 13.2$ were obtained. The correlation curve is shown in Figure 1.

### Evaluation of $T_3$-uptake

### Precision

Twenty determinations on each of three sera with different values of $T_3$-uptake were assayed to determine within-batch precision. The $T_3$-uptake values were then analysed in 20 separate batches to estimate the between-batch precision. Results are shown in Table 3.

### Carry-over and drift

Serum with a $T_3$-uptake value of 31% and another with a level of 56% were analysed alternatively in triplicate for a period of 5 hours. There was no indication of any significant carry-over or drift.

### Normal range

The normal range was determined on specimens from 309 adult blood donors. The mean $T_3$-uptake was 38.7% with a standard deviation of 3.2%. The distribution was Gaussian.

### General comments

Over a 9 month period the Star system was used daily for the estimation of large batches of serum $T_4$ and $T_3$-uptake. The analyser has proved to be simple to operate with reasonably short start-up and change-over times. Accuracy and precision of the $T_4$ assay and the precision of the $T_3$-uptake test were satisfactory. There were no problems

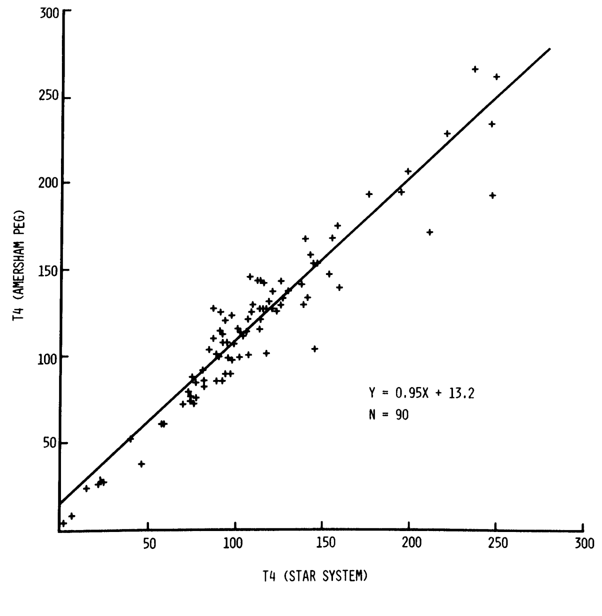

*Figure 1. Correlation of $T_4$ results between manual. (Amersham PEG) and automated (Star system) techniques.*

with carry-over or drift. Automation of these RIA assays has halved the man-hours normally required and eliminated the many errors which can occur in the manual analysis and clerical reporting of large batches of specimens.

One of the drawbacks of the system is that complete shutdown occurs when there is a printer failure. This fail-safe system is not essential when the analyser is connected to a laboratory computer. Another feature that could be improved is the sampler. A 40 specimen sampler is too small when large batches are being proceesed. Fewer plate changes would be required if the sampler size was increased to hold 60 or preferably 100 samples.

### Evaluation of the Olli C + D biochemical analyser after over a year of use in enzymology

**G. Baraton, D. Grafmeyer, A. Capuano, L. Richard and J. Sofia.**
*Laboratoire automatisé de Biochimie Clinique, (Professeur J. Bertrand), Hopital Edouard Herriot, Place d'Arsonval, 69374 Lyon Cedex 2, France*

To meet today's clinical requirements, clinical biochemistry laboratories must be increasingly automated. The required criteria for an automatic analyser are reliability; the ability to change chemical reactions easily and quickly; rapid determinations and low analysis cost.

It appears that the Olli C + D parallel analyser (Kone Oy instrument division Espoo, Finland) answers these criteria. A report on the Olli C system has already been published by Puuka and Pukka [1]. This evaluation, using the C + D system, deals with its intensive and daily use in enzymology over a period lasting more than one year.

### Description

The instrument is a parallel, multichannel, photometric analyser. The different phases of determination are performed in batches of 24 samples including blanks, standards and controls. The rate of analysis is 700 tests per hour with end point determinations of 360 kinetic measurements per hour – reaction time is 2½ minutes.

The analyser comprises three independent units, the sample diluter/processor, photometer (each controlled by microprocessor) and incubator. The sample change is performed by hand using disposable cuvettes in a thermostated block.

The Olli D Diluter measures 350 x 1100 x 740 mm and weighs 70 kg. It is made up of a dispensing tray with 24 cuvette blocks which fit in the Olli C photometer, eight reagent vessels, one block with 24 serum vials, an eight tip dispensing head and a digital display and keyboard. The diluter can be programmed by hand or by tape cassette for 30 analyses; it is possible to add serum and up to eight reagents distributed in one or several runs. Each analysis programme follows the sequence: 1. Attain reaction temperature (25, 30, 37°C). 2. Measure reagent (5 to 1000 μl by steps of 1μl) and position. 3. Measure and add sample. 4. Mix (5 to 180 seconds).

The photometer measures 330 x 520 x 740 mm and weighs 48 kg. It comprises a computer, a thermal printer and a thermostated measuring chamber (25°, 30°, 37°C). The light source is a zenon lamp. A quartz optic fibre system allows the simultaneous measurement of 24 cuvettes. Filters have a bandwidth of 8 to 15 nm. The smallest readable volume is 500 μl for end point determinations, and 300 μl for kinetic determinations.

The photometer can be programmed by hand or by tape cassette for 15 analyses which may be made in end point mode, with or without blank measurement, or in kinetic mode, with or without reagent or serum blanks. Kinetics determinations are made using linear regression of 12 to 24 measurements in 1 to 10 minutes. The results are printed with the name and units of the programmed analysis. Error messages are printed; eg, if the chosen initial absorbance limits have been exceeded or if there is non-linearity of reaction. In addition, it is possible to have printed the twelve or 24 absorbance measurements used for the calculation of the activity of each determination.

The Olli incubator can handle four thermostated blocks. Each block contains one heating element and temperature regulation is maintained via a water circulating system. The incubation period is determined by a timer and each block incubation time is terminated with an audible alarm.

### Material and methods

The evaluation was carried out according to the rules suggested by the French Society for Clinical Biology (SFBC) [2, 3].

### Dilution and measurement

Accuracy, within-block and day-to-day, was tested with a solution of drawing ink (Mars 745 from Staedler Germany) prepared as follows: 1.7 ml of ink as supplied was dissolved by gentle stirring in 1000 ml of distilled water and filtered in a buchner funnel through silicone paper. The solution was preserved with Merseptyl and stored at +4°C. Dilutions were made by the Olli D processor into Olli arcylic cuvettes and absorbances were read on the Olli C photometer at 405 nm. Measurements were verified by manual dilution followed by a reading using a Miniken spectrophotometer (Coultronic).

### Control of temperature

The control of the temperature variations between 25 and 37°C was tested by measuring the absorbance of a paranitrophenol solution, (15 mg in 1000 ml of Tris HCL 0.2 M, pH 6.8) at 405nm, Naudin *et al* [4]. The absorbances obtained at the chosen temperature were compared with those obtained using four thermostated analysers; Miniken (Coultronic), Cobas BIO (Roche,) PA 800 (Vitratron), ACP 5040 (Eppendorf). On the Olli C, the time needed to reach the set temperature of 30°C or 37°C with an analysis volume of 550 μl

